# Characterizing and explaining spatio-temporal variation of water quality in a highly disturbed river by multi-statistical techniques

**DOI:** 10.1186/s40064-016-2815-z

**Published:** 2016-07-26

**Authors:** Jianfeng Liu, Xiang Zhang, Jun Xia, Shaofei Wu, Dunxian She, Lei Zou

**Affiliations:** 1 State Key Laboratory of Water Resources and Hydropower Engineering Science, Wuhan University, Wuhan, 430072 China; 2 Hubei Provincial Collaborative Innovation Center for Water Resources Security, Wuhan University, Wuhan, 430072 China

**Keywords:** Water quality, Trend, Spatial autocorrelation, Climate variables, Land use, Water quality management

## Abstract

Assessing the spatio-temporal variations of surface water quality is important for water environment management. In this study, surface water samples are collected from 2008 to 2015 at 17 stations in the Ying River basin in China. The two pollutants i.e. chemical oxygen demand (COD) and ammonia nitrogen (NH_3_-N) are analyzed to characterize the river water quality. Cluster analysis and the seasonal Kendall test are used to detect the seasonal and inter-annual variations in the dataset, while the Moran’s index is utilized to understand the spatial autocorrelation of the variables. The influence of natural factors such as hydrological regime, water temperature and etc., and anthropogenic activities with respect to land use and pollutant load are considered as driving factors to understand the water quality evolution. The results of cluster analysis present three groups according to the similarity in seasonal pattern of water quality. The trend analysis indicates an improvement in water quality during the dry seasons at most of the stations. Further, the spatial autocorrelation of water quality shows great difference between the dry and wet seasons due to sluices and dams regulation and local nonpoint source pollution. The seasonal variation in water quality is found associated with the climatic factors (hydrological and biochemical processes) and flow regulation. The analysis of land use indicates a good explanation for spatial distribution and seasonality of COD at the sub-catchment scale. Our results suggest that an integrated water quality measures including city sewage treatment, agricultural diffuse pollution control as well as joint scientific operations of river projects is needed for an effective water quality management in the Ying River basin.

## Background

Rivers are the important source of fresh water for agriculture, industry, drinking supplies, and for recreational, navigational and hydropower activities. It offers a wide range of habitats for the aquatic flora and fauna (Varol et al. 2012). In an undisturbed river, the chemical composition of the water varies with time and space because of natural factors like climate and topography. Impairment of a river’s water quality because of anthropogenic activities such as disposal of wastewater, transfer of runoff from disturbed land, industrialization can cause major changes in water quality that in turn affect the human benefits and ecosystem services (Singh et al. 2004). It has been reported that about 54 % of the lakes in Asia are eutrophic, followed by Europe (53 %), North America (48 %), South America (41 %) and Africa (28 %) (Nyenje et al. 2010). Thus, the assessment of water quality is a major concern for an effective river management, especially in densely populated regions (Vega et al. 1998). In North America, the strategies for improving surface water quality have been initiated in the early 1970s followed by Europe in the 2000s (Hawkins 2015; Hering et al. 2010). In China, a national water pollution control act, Major Science and Technology Program for Water Pollution Control and Treatment was initiated in 2009 with the primary target of reducing the chemical oxygen demand (COD) and ammonia nitrogen (NH_3_-N) levels in surface water (Li et al. 2014). Considering the complex variation in water quality across time and space, an effective management of the river water quality requires two key types of information (1) spatial and temporal characteristics of the pollutants, and (2) information about the driving factors influencing the water quality, which have been a core task of water environment research around the world (e.g., Mostafaei 2014; Ogwueleka 2015; Phung et al. 2015).

The examination of long-term water quality variation by utilization of the trend detection technique is an effective way to derive potential water quality problems. Bouza-Deaño et al. (2008) applied the non-parametric Mann–Kendall Test to analyze the trend of nearly 34 physical–chemical variables in the Ebro River (Spain) and found that the water quality variation over time is due to decreasing phosphate concentrations and elevated pH levels. Further, Johnson et al. (2009) showed the impact of flow-adjusted pollutants (such as total suspended solids, total phosphorus, and orthophosphorus) in the Minnesota River (USA) using the non-parametric (Seasonal Kendall Test) and parametric (QWTREND) statistical techniques. Cluster analysis (CA), an unsupervised pattern recognition technique, has been widely used to identify the variation in hydro-chemical composition among seasons or among stations, which is useful for evaluating the contribution of point and non-point source pollution and establishing corresponding load reduction goals (Ouyang et al. 2006). In Kaduna River, Nigeria, Ogwueleka (2015) applied CA to group the yearly data in two seasons on the basis of seasonal variation in water quality parameters. However, previous studies usually analyzed the seasonal variation in hydro-chemical composition simply with spatially averaged water quality data, but no extensive analysis has been made towards identifying the seasonality of specific pollutant concentrations such as COD and NH_3_-N, which is essential for the control of primary pollutants in many seriously polluted rivers.

Furthermore, many authors have attempted to relate the spatial and temporal patterns of water quality variables with underlying causes such as climate change, catchment characteristics and anthropogenic activities using spatial autocorrelation analysis, regression analysis and correlation analysis. In Han River Basin, South Korea, weak to moderate positive spatial autocorrelation of water quality parameters was detected by using the Moran’s index, which Chang (2008) attributed to spatial heterogeneity in local water quality management, land use and geology. However, the seasonal variation in the spatial structure of water quality has not been explicitly analyzed in the past. In the New River, USA, Murphy et al. (2014) established significant negative linear regression equations between river discharge and four water quality parameters, and regarded the dilution of finite contaminant supply as the cause. With the advancement in remote sensing and Geographic Information System (GIS), correlation analysis has been increasingly applied to explore the influence of land use types on spatial pattern of water quality at different scales (Haidary et al. 2013; Lindell et al. 2010). Nevertheless, to our knowledge, rare studies are attempted on the analysis of land use types for explaining the seasonal variations in surface water quality.

In purview of the above-mentioned concerns, this paper is focused on revealing the complex relationships between the water quality and the related driving factors (climate variables, land use and water quality management) in the Ying River basin in China. Especially, the underlying influences of land use on seasonal pattern of water quality are attempted in this study. The chosen Ying River in China has witnessed the occurrence of five serious water pollution incidents, and hence needs restoration practices. Some studies have been attempted in local regions to investigate the specific toxic pollution (Fu et al. 2014). However, a more detailed comprehensive study is needed to understand the water quality changes in the basin. In this study, several statistical analysis techniques such as cluster analysis, seasonal Kendall test, Moran’s Index are performed for identifying the seasonal and inter-annual pattern in the water quality as well as to understand the latent spatial structure of the dataset. Further, the relationships between the specific pollutants with the climatic variables, land use and local water quality management are explored through regression and correlation analysis. The main goals of this research are to provide a comprehensive characterization of the water quality evolution in the typically disturbed Ying River basin and give scientific references for the implement of effective water pollution prevention in the future.

## Methods

### Study area

**Fig. 1:**
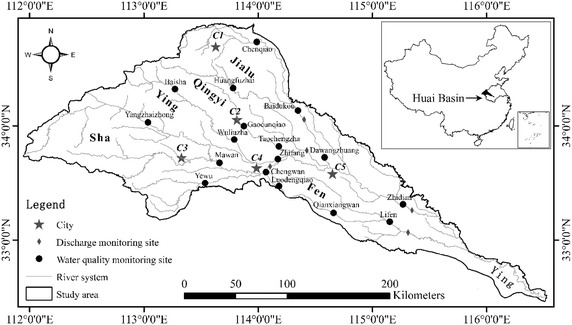
Location of water quality monitoring sites and flow monitoring sites in the study area

The Ying River is the largest tributary of the Huai River and considered as one of the most polluted river in China (Fig. 1). The upper Ying River emerges from the eastern foothills of the Funiu Mountain and in the way joined by the tributaries Qingyi, Sha, Jialu and Fen. The mainstream of the Ying River has a length of 624 km and drains a catchment with an area of 40,000 km^2^. The catchment is located between 111°54′E–116°33′E and 33°26′N–34°55′N coordinates and considered under subtropical-temperature climatic zone. The average annual rainfall for the catchment is approximately 770 mm, receiving 63 % of rainfall in the months of June to September. There is a pattern of increasing rainfall throughout the catchment in the downstream direction. The western mountains are the main source of summer rainstorms that pose a risk of flooding. In the other areas, the water from sewage system and farmlands irrigation contributes to the dry season flow. In order to deal with the issues of flood and drought sluices and dams are widely used in the area.

The five major urban cities of Zhengzhou (*C1*), Xuchang (*C2*), Pingdingshan (*C3*), Luohe (*C4*) and Zhoukou (*C5*) contribute large volumes of domestic and industrial wastewater to the river. The Ying River catchment accounts for only 14 % of the total drainage area of the Huai River Basin but is responsible for more than one-third of the two main pollutants, COD and NH_3_-N in the river (Zhang et al. 2013), which can disturb the ecological balance and deteriote human health. Even though the pollution events are not severe, but are serious enough to significantly impact ecological and human life on a long term basis.

### Data description

Weekly COD and NH_3_-N water quality data for 17 stations for the period 2008–2015 are obtained from the Department of Environmental Protection of Henan Province and the Huai River Water Resources Protection. The parameters COD and NH_3_-N are chosen as they are recognized as two most serious pollutants and found dominant in the Ying River system. Mean monthly water quality data over the period collected from all the stations are used for the purpose of cluster analysis. The daily river discharge and temperature time series for the period 2009–2010 for 5 stations, provided by Huai River Water Resources Commission Hydrology Bureau is used to depict relationship with the COD and NH_3_-N. The annual wastewater production and treatment data are obtained from Ministry of Environmental Protection of the People’s Republic of China (http://www.zhb.gov.cn).

Land use data of 2010, drainage network and digital elevation model (DEM) data are obtained from the Resources and Environmental Sciences Data Center, Chinese Academy of Sciences (RESDC, http://www.resdc.cn). Land use is classified into four major land types: urban, farmland, forest, and open water. To investigate the influence of land use on COD and NH_3_-N and their variability, the outlets of each sub-catchment are set at the monitoring stations, while the elevation and drainage network data are used for catchment delineation.

### Statistical analysis

Cluster analysis is used in this study to group the samples according to the seasonal pattern of pollutant concentrations. It is an unsupervised pattern recognition technique and is utilized to classify the objects based on similarity of their intrinsic structure without making a priori assumptions about the data (Vega et al. 1998). In this study, the monthly data from each station is normalized and then cluster analysis is performed using the Ward’s method, which is found useful for quantifying the proximity between samples (Juahir et al. 2010). The linkage distance represented on the y-axis is rescaled to a standard range of 0-25 for the sake of representation.

The seasonal Kendall test is a non-parametric test and is used to detect the inter-annual trends of COD and NH_3_-N. Considering the seasonality of the water quality, this test computes the Man-Kendall test for each identified season separately, and then combines the results (Helsel and Frans 2006). For estimating the magnitude of the trend, Sen’s slope method is used, which is the median of $\frac{{x_{ig} - x_{ih} }}{g - h}$ for all ($x_{ig}$,$x_{ih}$) pairs, where $x_{ig}$ means the pollutant concentration in the *i*th month and *g*th year (Kahya and Kalaycı 2004).

Moran’s $I$ (Moran 1948), a spatial autocorrelation indicator, is used to diagnose the spatial dependence of water quality and the related trend. The statistic can be calculated as:

1 $$I = \frac{n}{{\mathop \sum \nolimits_{i = 1}^{n} \left( {X_{i} - \overline{X} } \right)^{2} }}\frac{{\mathop \sum \nolimits_{i = 1}^{n} \mathop \sum \nolimits_{j = 1}^{n} W_{ij} \left( {X_{i} - \overline{X} } \right)\left( {X_{j} - \overline{X} } \right)}}{{\mathop \sum \nolimits_{i = 1}^{n} \mathop \sum \nolimits_{j = 1}^{n} W_{ij} }}$$

where $n$ is the number of stations, $X_{i}$ and $X_{j}$ refer to water quality index in stations $i$ and $j$ respectively, $\overline{X}$ is the mean value of water quality index, and $W_{ij}$ is a distance weight for the interaction between stations $i$ and $j$. The value Moran’s $I$ ranges from −1 and 1 that represents perfect negative or positive autocorrelation respectively, and no autocorrelation when it is zero (Tu and Xia 2008). Furthermore, local Moran’s $I_{i}$ (Anselin 1995), is also applied to identify the significant spatial clusters or outliers that help in the selection of stations, which contribute most to overall pattern of spatial dependence. The local Moran’s $I_{i}$ is calculated as:

2 $$I_{i} = \frac{n}{{\mathop \sum \nolimits_{j = 1}^{n} W_{ij} }}\frac{{\mathop \sum \nolimits_{j = 1}^{n} W_{ij} \left( {X_{i} - \overline{X} } \right)\left( {X_{j} - \overline{X} } \right)}}{{\mathop \sum \nolimits_{j = 1}^{n} \left( {X_{j} - \overline{X} } \right)}}$$

The local spatial patterns are classified as “*HH*”, “*LL*”, “*LH*” or “*HL*” (Anselin 1996). The “*HH*” or “*LL*” pattern indicates that the particular location is significantly having high or low values respectively, while the “*LH*” and “*HL*” represents the outliers surrounded by the high or low values, respectively (Zhai et al. 2014).

To identify the influence of discharge and temperature on pollutants concentration, ordinary least square (OLS) multiple linear stepwise regression is performed. The significance of estimated coefficients and regression model are tested through *t* test and *F* test (*F*_*sig*_) respectively. The goodness of fit of the regression models are provided by adjusted coefficient of determination (*R*_*adj*_^2^). The multi-collinearity between the independent variables is estimated by using the maximum variance inflation factor (*VIF*) and maximum condition index (*Cl*). The regression model exhibits multi-collinearity when the condition satisfy *VIF* > 10 or *Cl* > 30 (O’brien 2007; Velleman and Welsch 1981). Additionally, simple correlation analysis is used to explore the influence of land use on spatial distribution of pollutant concentrations.

## Results

### Temporal variation of water quality

#### Seasonal variation

**Fig. 2:**
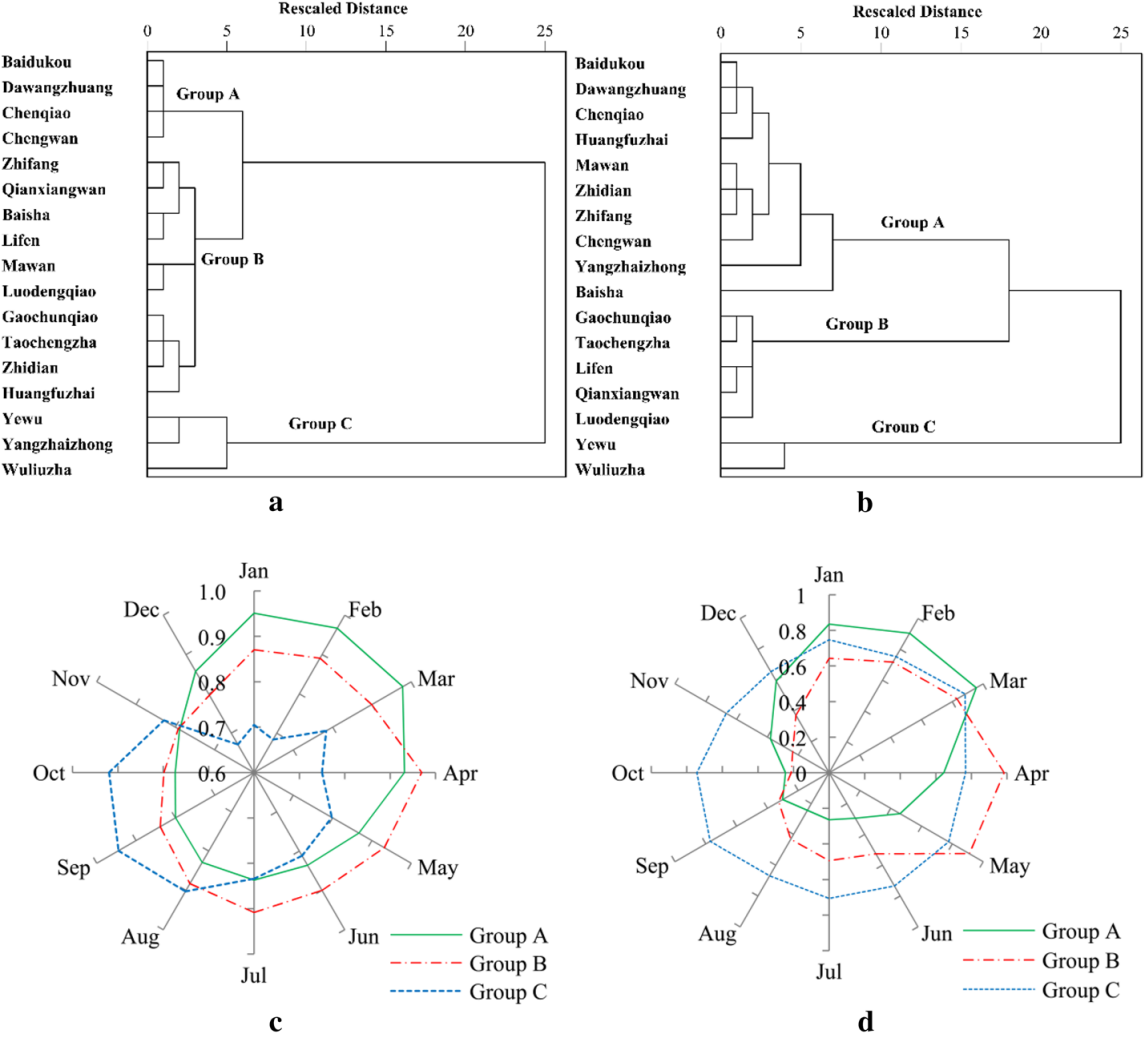
Clustering of monitoring sites according to seasonal variation of COD concentration (**a**) and NH_3_-N concentration (**b**), and the mean of normalized concentrations in COD groups (**c**) and NH_3_-N groups (**d**)

The 17 monitoring stations are grouped into three statistically significant clusters for characterizing the seasonal variation of COD and NH_3_-N (Fig. 2). Group A is characterized by high pollution of COD and NH_3_-N during the low flow winter months while low concentration in the high flow autumn months. Group B is showing a peak pollution of COD and NH_3_-N concentration during the pre-flood season in April and May and low concentration in autumn months. Group C indicates a highest pollution of COD during the period of late summer to early autumn and has the lowest concentration during the winter, while NH_3_-N shows a little change in concentration for the period under investigation. It is evident from the figure that for COD more than half of the stations are grouped into group B, while for NH_3_-N most of the stations are found in group A. In addition, NH_3_-N generally shows a larger variability than COD. These results suggest that the contribution of nonpoint source for COD are generally higher than for NH_3_-N. Only few stations are found in group C and mostly from western headwater zones.

#### Temporal trend of water quality

**Fig. 3:**
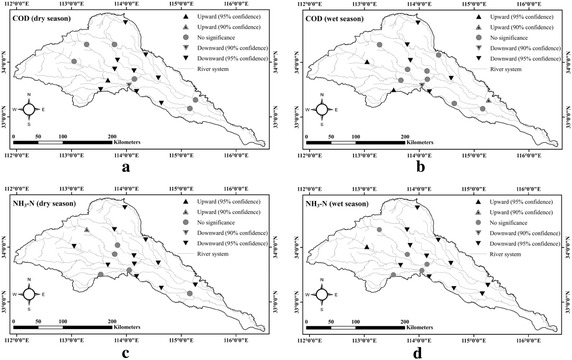
Temporal trends in water quality for **a** COD in dry season, **b** COD in wet season, **c** NH_3_-N in dry season, and **d** NH_3_-N in wet season over the period 2008–2015

The seasonal Kendall test is used to assess the water quality trend in the dry (November to May) and wet (June to October) seasons during the period of 2008–2015 at 17 stations (Fig. 3). For COD, 9 stations, mostly located in Jialu, Qingyi and Fen rivers, indicate a significant decreasing trend during the dry season with an exception of station Mawan located in the downstream of the Pingdingshan City, which is showing an increasing trend. In the wet season, a decreasing trend is observed from the analysis at 5 stations located in the downstream region in the cities Zhengzhou, Xuchang and Luohe, while a significant increasing trend is observed at two stations on the upper Sha River. For NH_3_-N, almost all the stations on Jialu, Qingyi and Fen rivers indicate an increasing trend during both the dry and wet seasons. On the other hand, a significant NH_3_-N concentration is found at upper Sha River during wet season.

### Spatial trend of water quality

**Fig. 4:**
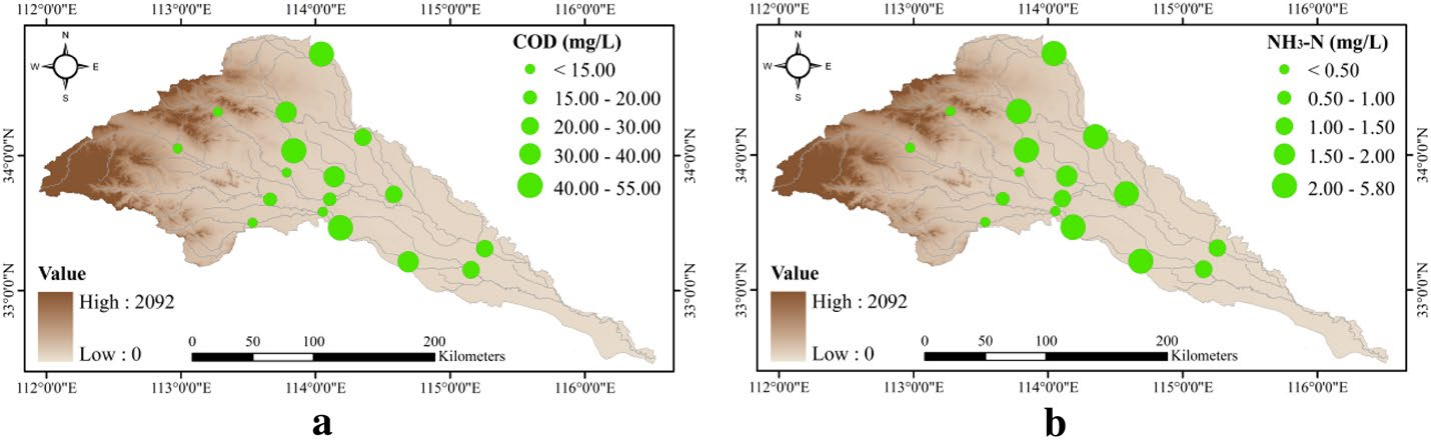
Spatial trends of water quality in the Ying River basin for **a** COD and **b** NH_3_-N

In general, COD and NH_3_-N have displayed similar spatial patterns, with the lower concentrations on the western Sha and upper Ying rivers and higher concentrations in the Jialu, Qingyi, and Fen rivers. In the less polluted Sha River, water quality is found good in the forested headwaters, but started deteriorating in the downstream direction where anthropogenic activities are found dominant. In contrast, in highly polluted Jialu, Qingyi and Fen rivers, water quality is found worst in the urbanized headwaters and showing a significant improvement in lower reaches. In the mainstream Ying River, water quality is generally good in the upper reaches and deteriorates in the lower parts, where it receives pollutants from the incoming tributaries (Fig. 4).

### Spatial autocorrelation of water quality concentration and trend

Spatial association of pollutant concentrations and trend are explored for both dry and wet seasons as shown in Table 1. A highly significant positive spatial autocorrelation (*α* < 0.01) is found for NH_3_-N in the dry season, suggesting that geographically neighboring stations have similar levels of NH_3_-N concentration. On the other hand, the Moran’s $I$ value is showing a weak autocorrelation at a significance level of *α* < 0.1 in the wet season, which suggests a localization in water quality. Similarly, high and weak positive spatial autocorrelations are obtained for the NH_3_-N trend in the dry and wet seasons respectively, implying similar NH_3_-N trends in neighboring stations. This difference in spatial autocorrelation between seasons might be ascribed to flow regulation and monsoon rainfall. Non-significant positive Moran’s $I$ values (*α* > 0.1) are detected for COD concentration for both the seasons, which might be attributed to the spatial heterogeneity in pollutant loads and local water quality management strategies.

**Table 1:** Spatial autocorrelation of water quality and water quality trend in dry and wet seasons

	COD concentration		NH_3_-N concentration		Sen’s slope of COD		Sen’s slope of NH_3_-N	
	Dry season	Wet season	Dry season	Wet season	Dry season	Wet season	Dry season	Wet season
Moran’s *I*	0.09	0.05	0.29	0.20	0.01	−0.08	0.26	0.19
*Z* Score	1.13	0.84	2.58^**^	1.91^*^	0.45	−0.17	2.37^**^	1.86^*^

Superscripts * and ** mean the element has a significance level of *α* < 0.10 and *α* < 0.01 respectively

Furthermore, Local spatial autocorrelation of pollutant concentrations and trend are detected using local Moran’s *I*_*i*_ (Table 2). Yewu station is showing an outlier for COD in both seasons, while Wuliuzha station is found to be an outlier for NH_3_-N in the dry season. These results imply there is a lower concentration of COD or NH_3_-N than the concentration obtained at the surrounding stations. In the dry season, cluster centers with high NH_3_-N concentration are found at Dawangzhuang station located in the downstream of Jialu River and at Qianxiangwan station located in the midstream of Fen River in both the seasons. These regions can be considered as significantly high NH_3_-N pollution zones. On the other hand, remarkable improvement in water quality is detected at Qianxiangwan station as a cluster center with large COD slope in the dry season and large NH_3_-N slope in both the seasons. The Dawangzhuang station is found to be a cluster center with large NH_3_-N slope in the dry season.

**Table 2:** The level of significance for local spatial association analysis for water quality and trend

Station	Significance level of COD concentration		Significance level of NH_3_-N concentration		Significance level of Sen’s slope of COD		Significance level of Sen’s slope of NH_3_-N	
	Dry season	Wet season	Dry season	Wet season	Dry season	Wet season	Dry season	Wet season
Chenqiao	0.33	0.29	0.35	0.88	0.90	0.96	0.58	0.99
Huangfuzhai	0.30	0.23	0.71	0.97	0.62	0.67	0.91	0.90
Baisha	0.24	0.26	0.23	0.39	0.58	0.49	0.46	0.42
Yangzhaizhong	0.86	0.72	0.60	0.40	0.60	0.45	0.48	0.44
Yewu	*0.06 (LH)*	*0.09 (LH)*	0.17	0.29	0.67	0.60	0.33	0.28
Mawan	0.38	0.42	0.23	0.23	0.97	0.92	0.28	0.23
Luodengqiao	0.24	0.26	0.23	0.39	0.49	0.38	0.35	0.51
Lifen	0.87	0.91	0.84	0.81	0.58	0.70	0.80	0.83
Gaocunqiao	0.19	0.21	0.92	0.99	0.31	0.29	0.51	0.80
Taochengzha	0.48	0.56	0.93	0.86	0.99	0.55	0.89	0.97
WuLiuzha	0.72	0.70	*0.09 (LH)*	0.16	0.77	0.87	0.32	0.36
Zhifang	0.81	0.86	0.99	0.92	0.44	0.55	0.63	0.62
Dawangzhuang	0.79	0.95	*0.06 (HH)*	0.67	0.71	0.56	*0.05 (LL)*	0.21
Baidukou	0.88	0.91	0.71	0.86	0.91	0.89	0.64	0.67
Chengwan	0.53	0.41	0.41	0.78	0.84	0.85	0.40	0.57
Qianxiangwan	0.33	0.40	*0.03 (HH)*	*0.00 (HH)*	*0.00 (LL)*	0.72	*0.00 (LL)*	*0.02 (LL)*
Zhidian	0.87	0.91	0.84	0.81	0.58	0.70	0.80	0.83

A statistic in italic mean the station is the center of a cluster, or an outlier, for water quality statistics with significance level less than 0.1. *LH*, *HH* and *LL* represent “low–high”, “high–high” and “low–low” spatial pattern

### Factors influencing water quality

#### Relation between water quality and climatic variables

Multiple regression analysis between weekly log-transformed pollutant concentration, water temperature (*T*) and water discharge (*Q*) during the period of 2009 to 2010 is conducted for the downstream stations of Sha (Chengwan), Fen (Lifen), Jialu (Baidukou) tributaries and the mainstream stations (Zhifang and Zhidian). As shown in Table 3, the models that passed the *F*-test with 0.05 significant level and have no serious colinearity between the water discharge and temperature (*VIF* < 5 and *Cl* < 10) are considered best. Following the criteria, no significant regression model could be established at Lifen station for NH_3_-N. Generally, *T* is negatively associated with NH_3_-N at most of the stations while fewer stations in case of COD. *Q* tends to show varying influence on pollutant concentration among stations. At Baidukou station, both *T* and *Q* show negative association with pollutant concentrations. However, for all the other stations, the influence of *T* and *Q* on pollutant concentration tends to be complex. Also positive correlations are obtained for *Q* with COD or NH_3_-N at two stations, namely, Zhifang and Chengwan. NH_3_-N regression models are showing better fitness function than COD with the higher values of *R*_*adj*_^2^, which could be attributed to that NH_3_-N is more correlated with temperature-dependent in-stream biochemical process than COD. However, the fitting degrees of the regression models is generally low (*R*_*adj*_^2^ ≤ 0.5) probably due to the disturbance of sluices and dams regulation on river flow.

**Table 3:** Regression analysis of COD and NH_3_-N pollutants

Station	COD							NH_3_-N						
	*Const*	*β*_*T*_	*β*_*Q*_	*F*_*sig*_	*R*_*adj*_^2^	*VIF*	*Cl*	*Const*	*β*_*T*_	*β*_*Q*_	*F*_*sig*_	*R*_*adj*_^*2*^	*VIF*	*Cl*
Baidukou	***1.5627***	**−** ***0.0020***	**−** ***0.0017***	0.01	0.28	1.01	4.89	***1.0625***	**−** ***0.0104***	**−** ***0.0078***	0.01	0.40	1.01	4.89
Zhifang	***1.2708***	**−** ***0.0014***	*0.0015*	0.01	0.17	1.08	4.09	*0.2282*	**−** ***0.0067***	−*0.0125*	0.01	0.15	1.08	4.09
Chengwan	***1.2321***	−0.0032	–	0.60	0.04	1.12	4.33	0.1321	**−** ***0.0391***	***0.001***	0.01	0.38	1.12	4.87
Lifen	***1.3748***	–	**−** ***0.0012***	0.02	0.01	1.00	2.94	–	–	–	–	–	–	–
Zhidian	***1.3933***	−0.0030	**−** ***0.0004***	0.01	0.17	1.24	4.85	***0.603***	**−** ***0.0190***	**−** ***0.0005***	0.01	0.50	1.24	4.85

Estimated coefficients (Const: constant intercept, $\beta_{T}$: water temperature, $\beta_{Q}$: water discharge) in bolditalic or italic mean 0.05 or 0.10 significant level of *t* test). $F_{sig}$ and $R_{adj}^{2}$ are the significant level of *F* test and determination coefficient for the estimated model. *VIF* and *Cl* are variance inflation factor and maximum condition index

#### Relation between water quality and land use

The Pearson correlation coefficient is used to explore the influence of land use on the pollution level and variability (*Cv*) of water quality (Table 4). At the sub-catchment scale, urban land use is showing a highly significant positive correlation with the COD and NH_3_-N concentrations. Inversely, forest cover indicates a significantly high negative correlation with the COD and NH_3_-N concentrations. The influence of farmland on water quality is found to be negative, while it is very low in case of urban land use. Compared at a sub-catchment scale, correlation between land use and water quality concentration at the 100 m buffer scale is found relatively weak, which is similar to the previous studies (Meynendonckx et al. 2006). On the other hand, the variability of COD is found positively associated with the forestland in both sub-catchment and buffer scales, while negatively associated with the farmland at sub-catchment scale. However, there is no significant correlation found between the land use and NH_3_-N variability at both sub-catchment and buffer scales, implying that the seasonal NH_3_-N fluctuation is less sensitivity to terrestrial transport process.

**Table 4:** Pearson correlation coefficient (*r*) between land use categories and water quality parameters

Scale	Water quality	*r*_*1*_ (between land use and pollutant concentration)				*r*_*2*_ (between land use and *Cv* of pollutant concentration)			
		Urban land	Farmland	Forest	Water	Urban land	Farmland	Forest	Water
Sub-catchment	COD	0.89**	0.50*	−0.63**	−0.51*	−0.45	−0.60*	0.64**	0.44
	NH_3_-N	0.77**	0.52*	−0.65**	−0.26	−0.36	0.10	−0.02	−0.01
100 m buffer	COD	0.67**	0.58*	−0.53*	−0.67**	−0.37	−0.49	0.56*	0.47
	NH_3_-N	0.17	0.50*	−0.51*	−0.48	−0.14	0.009	0.01	0.14

“*” is significant at α ≤ 0.05and “**” is significant at α ≤ 0.01

**Fig. 5:**
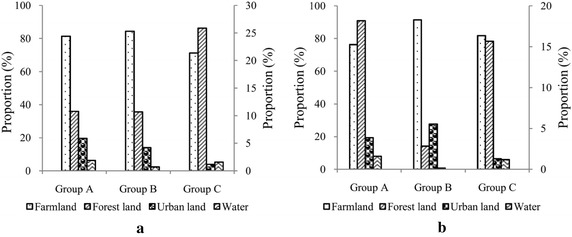
Mean proportion of land use types in sub-catchments of stations within the COD groups (**a**) and NH_3_-N groups (**b**) identified by cluster analysis in Fig. 2. Farmland is on the primary axis and the others are on the secondary axis

The average composition of land use in the sub-catchment scale for the groups in Fig. 2 is calculated and shown in Fig. 5. In COD groups, there is a gradual decrease in the urban land proportion occurred from group A to group B and then group C, implying a decrease in the influence of urban sewage. Group B is characterized by the highest farmland proportion, representing the predominant influence of the agricultural pollution, while group C is under the lowest level of anthropogenic influence due to the highest forestland proportion. The results suggest that land use could be well used for predicting the seasonal pattern of organic pollutants in surface water system. For NH_3_-N, a non-significant correlation between land use and NH_3_-N variability is obtained (Table 4). Generally, group B is characterized by the highest composition of urban and farmland proportions, which imply a combined influence of the urban sewage and agricultural pollution. The lowest proportion of farmland proportion is found in group A, showing a predominant influence of urban sewage.

#### Pollutant load change

**Fig. 6:**
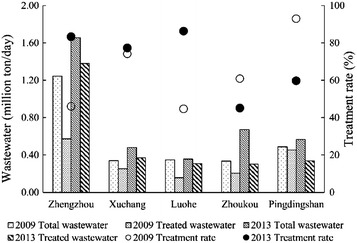
Changes in wastewater production, treatment capacity and treatment rate in five cities between 2009 and 2013

Generally, reduction of point source pollution load leads a year-round improvement of water quality while the reduction of non-point source load provide an improvement in water quality during the wet season only. Therefore, the observed trend of water quality can be ascribed to the changing pollution sources in response to the management actions. As shown in Table 5, total 9 stations indicate a reduction in point source COD load, while 6 stations exhibit an increasing trend of diffuse COD load. For NH_3_-N, significant reduction in point source load is found at 11 stations, while the change in diffuse load only exists at few stations. Further, the change in point source pollutant load in Ying River catchment is investigated by comparing the annual total municipal wastewater production and wastewater treatment quantity in the five cities for the periods 2009 and 2013. As shown in Fig. 6, Zhengzhou and Luohe indicate a substantial increase in the wastewater treatment rate that leads to a reduction in the untreated wastewater discharge. On the contrary, Zhoukou and Pingdingshan cities show a decreased wastewater treatment rate and therefore an increasing amount of untreated wastewater is released into the river. Finally, in Xuchang City, the improvement of wastewater treatment rate is very weak as the wastewater treatment capacity is in synchronization with wastewater production.

**Table 5:** Identifying result of pollutant load change

Change of pollutant load	Increase from point source (UU, UN, UD)	Decrease from point source (DU, DN, DD)	Increase from nonpoint source (NU, DU, DN)	Decrease from nonpoint source (UN, UD, ND)
Number of sites (COD)	1	9	6	2
Number of sites (NH_3_-N)	1	11	2	3

“U” means significant upward trend, “D” means significant downward trend, “N” means no significant trend. “UD” means significant upward trend in dry season and downward trend in wet season, and other combination patterns were similar

## Discussion

### Climatic variables

The negative correlation between water temperature and pollutant concentrations in regression models suggests that there is an enormous influence of in-stream biological activities on water quality. Similar findings were also reported by Mietto et al. (2015), who indicated that higher temperature during the warm season facilitates microbial degradation of water contaminants and therefore effectively improves the water quality. In addition, aquatic plants can also affect the cycling of nutrients through uptake and temporary storage of nutrients during warm growing season (Clarke 2002). However, the correlation is found very weak for COD in tributary Sha and Fen and the lower mainstream possibly due to that high rainfall in flood season reduces the biochemical degradation efficiency of organic pollutants by shortening the hydraulic residence time (Poach et al. 2004). It is also seen that river discharge change resulting from monsoon climate exerts distinct influence on water quality among tributaries, suggesting a spatially varying composition of pollutant load. The Jialu tributary is found contaminated mainly by municipal wastewater from the upstream Zhengzhou region, as a result of which contamination concentrations are generally higher during the dry season and reduce greatly in rainy season due to the dilution effect. In Sha tributary and upper mainstream, rainfall-runoff pollution and eroded sediments due to the high flow exerts a negative influence on water quality in flood season (Park et al. 2011). The correlation between NH_3_-N and climatic variables is found nonsignificant in Fen tributary, which is possibly due to that perennial and unstable sewage discharge and excessive flow regulation weaken the predictability of river water quality.

Moreover, the spatial autocorrelation of water contaminants is found higher in the dry season possibly due to streamflow regulation and seasonal change of rainfall, which is rarely discussed in the previous studies (Chang 2008; Zhai et al. 2014). As a highly regulated river, the Ying River is found to be segmented into a number of dams and sluices that cause serious obstruction in the river flow. During the non-flood season when the dams are not operational, a high amount of contaminants is accumulated in the tributaries and results in high NH_3_-N pollution zones at the Dawangzhuang and Qianxiangwan stations. In the western upstream regions, many large sluices are closed to meet the water requirement, which could effectively improve the water environment capacity of upper reaches due to increased amount of water but cause an increase in water contaminants with reduced downstream flow velocity in the lower reaches (Zhang et al. 2010). In the rainy season, the relatively weak spatial dependence of water quality implies the local nonpoint source pollution and increased hydrological connectivity.

### Land use

While there are mixed findings on scale effect from the other studies (Sliva and Williams 2001; Tran et al. 2010), this study indicates that the land use at sub-catchment scale shows a better ability of explaining the spatial distribution of COD and NH_3_-N. High concentration and continuous discharge of city sewage from the urban areas plays a primary role in the deterioration of water quality. On the other hand, vegetation coverage is generally found promising for improving the water quality due to low pollutant load. Besides, the forestlands in the Ying River are found spatially distributed in the upstream steep hillsides, where the velocity of river flow is generally high that promotes the degradation of the pollutants (Kannel et al. 2007). The association between agritural activity and contaminant concentration is relatively weak, implying a spatially varying role in water quality deterioration (Tu and Xia 2008). In less-urbanized western regions, emissions from the livestock manure, fertilizers and pesticides in the runoff are usually the major factors for water quality deterioration. In contrast, the disturbances due to agricultural activities on water quality are found to be secondary in highly-urbanized areas, where industrial, commercial and residential lands are usually the major pollution sources. Therefore, a better understanding of the influence of agricultural pollution on water quality is needed at a local level.

Interestingly, the composition of land use is also found responsible for the seasonal variations in COD. Higher percentages of the urban land generally cause an increase in COD concentration in the dry winter, while higher forest lands with sporadic human activity disturbance causes an elevated COD level during the wet season. Unexpectedly, most rural areas report a high COD concentration in pre-flood months probably as the result of seasonal scale first flush phenomenon (Martin et al. 2014). Soller et al. (2005) suggested that the serious diffuse pollution is a function of storm intensity and long antecedent dry period. It might be the reason that the cumulative pollutants from the agricultural and residential lands are flushed down to the rivers due to some precipitation events in the initial period of rainy season and hence cause an increase in COD concentration. The results indicate that the priority for controlling the rural diffuse pollution should be given in pre-flood months. High values of the NH_3_-N levels are reported during the months of January to March at most of the stations, which imply that the NH_3_-N is more related to the in-stream hydrological regime and biochemical process.

### Water quality management

The significant improvement in water quality in Jialu, Qingyi and Fen rivers can be attributed to the efficient functioning of newly installed sewage treatment plants, which helps in reducing the pollutant emission from upper Zhenghou, Xuchang and Luohe cities. In addition, extensive local restoration projects including river dredging, riparian artificial wetland construction implemented in upper urban areas also play an important role in restoring local habitat and upstream retention (Hoffmann et al. 2011). However, the untreated sewage discharge is still the most important cause of water pollution in these areas. As the rapid development of urban and rural integration (Dai et al. 2015), rural non-point source pollution shows an upward trend in lower reaches of Jialu, Qingyi and Fen rivers, where comprehensive measures including riparian wetland and better farmland management should be adopted to improve local water environment (Dosskey et al. 2010). In western headwater regions, water quality is generally good due to less sewage discharge, while upward trend of water quality variables is found in the rainy season as a result of increasing soil erosion, instream sand excavation and agricultural development.

Although the water quality variability could be attributed to hydrological regime, land use and sewage disposal to some extent, some other potential risk factors appear to be critical for water quality management in the Ying River basin. Even though, the storm-water pollution from impervious surfaces (building sites, roads, parking lots, etc.) is increasing due to uncontrolled urban sprawl and high frequency hydrological events, the treatment of storm-water pollution is almost negligible, unlike in developed countries such as Australia and United States (Roy et al. 2008). Secondly, the population density has grown to nearly 900 persons/km^2^ in the study area, with more than half of the population residing in rural areas, where wastwater collection is usually difficult and not cost-effective. Therefore, decentralized treatment of household sewage is necessary in rural areas for local water quality management (Ichinari et al. 2008). In addition, unreasonable sluice regulation has proven to be a potential cause of increase in water pollution. Especially in pre-flood season, most dams and sluices in Ying River basin are opened to discharge stored water for flood control. The sudden release of accumulated pollutant concentrations in tributaries greatly destroys the water environment of lower trunk stream. Dam removal can help in providing an effective means for restoring river habitat in the developed areas (Foley et al. 2015). However, it cannot be applied to developing areas with rising demand for power and water. Therefore, considering the fact that water pollution level and seasonal variation pattern is different in the basin, scientific joint operation of water projects ameliorating the influence of upstream water on the downstream water quality and tributary water on the mainstream water quality could be provided as an effective solution for Ying River basin management.

## Conclusion

Effective basin water management requires a sound understanding of water pollution in rivers like Ying as the detection of water quality evolution and relevant contributing factors could provide a scientific support for water pollution control. The results show that:

(1) Three clusters characterized by different seasonal variation pattern were detected for both COD and NH_3_-N. Significant decrease in annual NH_3_-N concentration is found at more than half of the stations in both dry and wet seasons, while COD concentration decrease mainly during the dry season. Water quality is found to deteriorate in the western headwater reaches in the wet season.

(2) The seasonal fluctuation of water quality is closely related to the water temperature and discharge. Water temperature generally shows negative association with water quality variables, while river discharge exerts distinct influence on water quality among tributaries due to the spatially varying composition of pollutant load. In addition, seasonal change in spatial dependence of water variables is detected, which could be attributed to sluices and dams regulation and rainfall-runoff pollution.

(3) Land use at the sub-catchment scale provides a better explanation for spatial and temporal variation in COD and NH_3_-N. Generally, urban land and forestland are the two primary land use types responsible for the spatial distribution of COD and NH_3_-N. Further, the composition of Land use is found useful for explaining the seasonal variations in COD but not for NH_3_-N, suggesting that COD and NH_3_-N are more related to terrestrial transport process and in-stream factors, respectively.

(4) The inter-annual improvement of water quality in the dry season illustrates the effectiveness of urban water pollution control practices, while an upward trend of non-point source pollution is observed in some rural areas and western headwater regions. In addition, unreasonable regulation of sluices, urban runoff pollution and absence of rural wastewater treatment also pose a great threat to water quality. Thus, it can be concluded that comprehensive measures including sewage and stormwater treatment, agricultural pollution control, and scientific sluices regulation should be strengthened for water environment improvement in the highly disturbed Ying River basin.
